# Less sedentary time is associated with a more favourable glucose-insulin axis in obese pregnant women—a secondary analysis of the DALI study

**DOI:** 10.1038/s41366-020-0639-y

**Published:** 2020-07-13

**Authors:** Anna M. Dieberger, Gernot Desoye, Erwin Stolz, David J. Hill, Rosa Corcoy, David Simmons, Jürgen Harreiter, Alexandra Kautzky-Willer, Fidelma Dunne, Roland Devlieger, Ewa Wender-Ozegowska, Agnieszka Zawiejska, Annunziata Lapolla, Maria Grazia Dalfra, Alessandra Bertolotto, Sander Galjaard, Juan M. Adelantado, Dorte Møller Jensen, Lise-Lotte Andersen, Mette Tanvig, Peter Damm, Elisabeth Reinhardt Mathiesen, Frank J. Snoek, Judith G. M. Jelsma, Mireille N. M. van Poppel

**Affiliations:** 1 Department of Obstetrics and Gynaecology, Medical University of Graz, Graz, Austria; 2 Institute of Social Medicine and Epidemiology, Medical University of Graz, Graz, Austria; 3 Recherche en Santé Lawson SA, Bronschhofen, Switzerland; 4 Lawson Health Research Institute, London, ON N6C 2R5 Canada; 5 Institut de Recerca de l’Hospital de la Santa Creu i Sant Pau, Barcelona, Spain; 6 Department of Medicine, Universitat Autonoma de Barcelona, Barcelona, Spain; 7 CIBER Bioengineering, Biomaterials and Nanotechnology, Instituto de Salud Carlos III, Madrid, Spain; 8 Macarthur Clinical School, Western Sydney University, Sydney, Australia; 9 Gender Medicine Unit, Division of Endocrinology and Metabolism, Department of Medicine III, Medical University of Vienna, Vienna, Austria; 10 Galway Diabetes Research Centre and College of Medicine Nursing and Health Sciences, National University of Ireland, Galway, Ireland; 11 KU Leuven Department of Development and Regeneration: Pregnancy, Fetus and Neonate, Gynaecology and Obstetrics, University Hospitals Leuven, Leuven, Belgium; 12 Division of Reproduction, Poznan University of Medical Sciences, Poznan, Poland; 13 Universita Degli Studi di Padova, Padua, Italy; 14 Azienda Ospedaliero Universitaria Pisana, Pisa, Italy; 15 Department of Obstetrics and Gynaecology, Division of Obstetrics and Prenatal Medicine, Erasmus MC, University Medical Centre Rotterdam, Rotterdam, the Netherlands; 16 Steno Diabetes Center Odense, Odense University Hospital, Odense, Denmark; 17 Department of Gynaecology and Obstetrics, Odense University Hospital, Odense, Denmark; 18 Department of Clinical Research, Faculty of Health Sciences, University of Southern Denmark, Odense, Denmark; 19 Center for Pregnant Women with Diabetes, Departments of Endocrinology and Obstetrics, Rigshospitalet, Copenhagen, Denmark; 20 Department of Clinical Medicine, Faculty of Health and Medical Sciences, University of Copenhagen, Copenhagen, Denmark; 21 Department of Medical Psychology, Amsterdam University Medical Centres, Vrije Universiteit Amsterdam, Amsterdam Public Health Research Institute, Amsterdam, the Netherlands; 22 Department of Public and Occupational Health, Amsterdam Public Health research institute, Amsterdam UMC, Vrije Universiteit Amsterdam, Amsterdam, the Netherlands; 23 Institute of Sport Science, University of Graz, Graz, Austria

**Keywords:** Obesity, Gestational diabetes, Lifestyle modification

## Abstract

**Background/objectives:**

Obese pregnant women are at high risk of developing gestational diabetes mellitus (GDM), which might be reduced by sufficient physical activity (PA) and reduced sedentary time (ST). We assessed whether PA and ST are longitudinally associated with the glucose-insulin axis in obese pregnant women.

**Subjects/methods:**

In this secondary analysis of the DALI (vitamin D And Lifestyle Intervention for gestational diabetes mellitus prevention) study, pregnant women, <20 weeks gestation, with a pre-pregnancy body mass index (BMI) ≥ 29 kg/m^2^, without GDM on entry were included. Time spent in moderate-to-vigorous PA (MVPA) and ST were measured objectively with accelerometers at *<*20 weeks, 24–28 weeks and 35–37 weeks of gestation. Fasting glucose (mmol/l) and insulin (mU/l), insulin resistance (HOMA-IR) and first-phase and second-phase insulin release (Stumvoll first and second phase) were assessed at the same time. Linear mixed regression models were used to calculate between-participant differences and within-participant changes over time. Analyses were adjusted for gestational age, randomisation, pre-pregnancy BMI, education and age. MVPA, Insulin, HOMA-IR and Stumvoll first and second phase were log-transformed for analyses due to skewness.

**Results:**

232 women were included in the analysis. Concerning differences between participants, more ST was associated with higher fasting glucose (Estimate: 0.008; 95% CI: 0.002, 0.014), fasting insulin (0.011; 0.002, 0.019), HOMA-IR (0.012; 0.004, 0.021) and Stumvoll first and second phase (0.008; 0.001, 0.014 and 0.007; 0.001, 0.014). Participants with more MVPA had lower Stumvoll first and second phase (−0.137; −0.210, −0.064 and −0.133; −0.202, −0.063). Concerning changes over time, an increase in ST during gestation was associated with elevated Stumvoll first and second phase (0.006; 0.000, 0.011).

**Conclusions:**

As the glucose-insulin axis is more strongly associated with ST than MVPA in our obese population, pregnant women could be advised to reduce ST in addition to increasing MVPA. Moreover, our findings suggest that behaviour change interventions aiming at GDM risk reduction should start in early or pre-pregnancy.

## Introduction

Gestational diabetes mellitus (GDM), one of the most common pregnancy complications, occurs in about 6.1% (1.8–31.0%) of all pregnancies in Europe [1], with numbers growing steadily due to the continuous rise of obesity among pregnant women [2]. GDM is associated with a substantially elevated risk of adverse outcomes for mother and offspring including foetal overgrowth and increased caesarean section rates on the short term [3], and offspring obesity and development of type 2 diabetes in the mother on the long term [4].

Pre-conceptional obesity is the most important modifiable risk factor for developing GDM [5]. However, this factor cannot be addressed in prenatal care, as most women first get in contact with a health care professional when already pregnant. Therefore, other modifiable risk factors during pregnancy, such as physical activity and their association with GDM, need to be considered.

Physical activity in pregnancy has been shown to improve glucose uptake and reduce circulating insulin [6, 7]. In accordance with this, a meta-analysis showed that physical activity before and in pregnancy was associated with a significantly lower risk of developing GDM [8]. However, most included studies measured physical activity before or in early pregnancy, not considering that on average activity levels decrease with increasing gestation [9, 10]. These longitudinal changes of physical activity levels over the course of pregnancy might influence the association with glucose and insulin.

Furthermore, next to physical activity, too much sedentary behaviour has been identified as an independent risk factor for type 2 diabetes, cardiovascular disease and premature death outside of pregnancy [11–14]. However, the few studies assessing the relationship between sedentary behaviour and the glucose-insulin axis in pregnancy show inconsistent results [15], possibly due to heterogeneity in the definitions used and the measurement of sedentary behaviour. Objective measurements of both sedentary behaviour and physical activity at multiple time points in pregnancy are needed to gain more insight into how these two behaviours modify the glucose-insulin axis.

The vitamin D And Lifestyle Intervention for gestational diabetes mellitus prevention (DALI) study compared a lifestyle intervention, including the promotion of physical activity, to usual care as prevention for GDM [16]. While the interventions resulted in a reduction in sedentary time, an increase in moderate-to-vigorous physical activity (MVPA) and a reduction in neonatal adiposity, these lifestyle changes did not result in improvement in glucose or insulin concentrations or insulin resistance [17, 18]. However, differences in MVPA and sedentary time between the intervention groups were small. Therefore, analysing all participants as one cohort provides the opportunity to study a greater variation in activity levels. We hypothesise that reduced sedentary time and increased MVPA will improve the glucose-insulin axis. Previously reported improvements in physical activity and sedentary time in the DALI study were based on self-reported questionnaire data. For this secondary analysis, a sub-sample for which objectively measured accelerometer data are available, will be used to study physical activity levels more accurately.

In summary, the aim of this study is to investigate the longitudinal relationship between objectively measured physical activity levels and sedentary time and the glucose-insulin axis in obese pregnant women.

## Methods

### Participants

This is a secondary analysis of the DALI study, a multicentre randomised controlled trial. The DALI study was conducted between 2012–15 in nine European countries at eleven different sites (Austria, Belgium, Denmark (Odense, Copenhagen), Ireland, Italy (Padua, Pisa), Netherlands, Poland, Spain and United Kingdom) and was preceded by a pilot study. The study was registered under trial registration number ISRCTN70595832. Ethical approval was obtained from all local ethics committees and written informed consent was signed by all participants prior to data collection [16].

Pregnant women *<*20 weeks of gestation with a singleton pregnancy, aged ≥18 years with a pre-pregnancy body mass index (BMI) of ≥29 kg/m^2^ were asked to participate. All participants were screened for GDM before inclusion (*<*20 weeks), following the criteria of the International Association of Diabetes in Pregnancy Study Groups (IADPSG) [19] and were excluded if GDM was diagnosed. Other exclusion criteria encompassed pre-existing diabetes, chronic medical diseases, as well as abnormal calcium metabolism or calcium measurements in early pregnancy for the vitamin D trial [16]. For this study, only participants with objectively measured physical activity levels measured at least two out of three times were included to allow for longitudinal analyses.

Participants were randomised in the pilot trial to either a healthy eating (HE), a physical activity (PA) or a combined (HE&PA) intervention, in the lifestyle trial to HE, PA or HE&PA intervention or a control group, and in the vitamin D trial, to vitamin D supplementation with and without a HE&PA intervention or a placebo group with or without a HE&PA intervention. For this paper, data were analysed within an observational setting, combining all randomised participants from the pilot, lifestyle and vitamin D study into one cohort.

### Data collection

Data collection took place at three time points in pregnancy (*<*20 weeks, 24–28 weeks and 35–37 weeks) and at delivery. At each time point, women who had not developed GDM undertook a standardised 75 g oral glucose tolerance test (OGTT) after a night of fasting, with blood samples taken fasting and at 60 and 120 min after glucose ingestion. The blood samples were centrifuged and separate aliquots (1000 µl or 250 µl) were placed in microrack tubes. The samples were then stored at −20 °C or −80 °C until further analysed at the central trial laboratory in Graz, Austria. Glucose (mmol/l) was measured using the hexokinase method (DiaSys Diagnostic Systems, Holzheim, Germany) with a lower limit of sensitivity of 0.1 mmol/l. Insulin (mU/l) was quantified by a sandwich-immunoassay (ADVIA Centaur, Siemens Health Care Diagnostics Inc., Vienna, Austria) with an analytical sensitivity of 0.5 mU/l, intra-assay coefficient of variation of 3.3% to 4.6%, and inter-assay coefficient of variation of 2.6% to 5.9%. All assays were performed according to the instructions of the manufacturer.

Development of GDM over the course of pregnancy was assessed at 24–28 and 35–37 weeks, based on the IADPSG criteria: fasting venous plasma glucose ≥5.1 mmol/l and/or 1 h glucose ≥10 mmol/l and/or 2 h glucose ≥8.5 mmol/l. Homeostatic Model Assessment for Insulin Resistance (HOMA-IR) was calculated as (glucose_0_ × insulin_0_)/22.5 [20] and has been validated in pregnancy [21]. As a measure of beta cell function, first and second-phase insulin release were calculated, according to Stumvoll et al., as follows [22]: Stumvoll first phase = 1194 + 4.724 × insulin_0_ –117 × glucose_60_ + 1.414 × insulin_60_; Stumvoll second phase = 295 + 0.349 × insulin_60_ –25.72 × glucose_60_ + 1.107 × insulin_0_. Stumvoll first and second phase have not been validated in pregnancy.

Between the blood tests, anthropometric measurements and questionnaire data were gathered. Maternal age, ethnicity (Caucasian versus non-Caucasian), parity (multiparity versus nulliparity), marital status (with versus without partner), employment (yes/no), smoking status (yes/no), alcohol consumption (yes/no), and pre-pregnancy weight were assessed by questionnaire. Weight during pregnancy was measured at the three defined time-points using calibrated electronic scales (SECA Measure 888; 877). Maternal height was recorded on a stadiometer (SECA 206, SECA, Birmingham, UK) during the first visit. Pre-pregnancy BMI was calculated as pre-pregnancy weight (kg) divided by the square of height (m^2^). Information on offspring sex was obtained from medical records.

Physical activity was measured objectively by use of accelerometers (ActiGraph GT1M, GT3X+ or Actitrainer; Pensacola, Florida, USA) at *<*20 weeks, 24–28 weeks and 35–37 weeks [23]. Participants were instructed to wear the accelerometers during waking hours for three days on an elastic belt positioned over the right hip and to only remove the device while performing water-based activities such as swimming or showering. Time and reason of removal were recorded in an activity diary. Non-wear time was defined as periods with zero counts for at least 90 min [24]. A measurement was only deemed valid if the daily wear time was *>*480 min. For each time period three valid days had to be available. Then, the average number of minutes per day in sedentary (*<*100 counts/min), light (100–1951 counts/min) and moderate-to-vigorous physical activity (MVPA) (*>*1951 counts/min), were defined according to Freedson cut-off points [25]. Swimming time, as recorded in the activity diary, was added to minutes spent in MVPA [25, 26].

### Statistical analysis

Sociodemographic characteristics of all included participants were presented by mean and standard deviation (SD), median and interquartile range (IQR) or count and proportion and were compared to those excluded due to missing accelerometer data, using unpaired *t*-test or chi-square test. The outcome variables (fasting glucose, fasting insulin, HOMA-IR, Stumvoll first and second phase) and accelerometer variables (sedentary time, MVPA and wear time) are presented separately for each time point. Differences between time points were tested by paired sample t-tests. Variables that were not normally distributed were log-transformed prior to analyses.

The main analysis was performed using longitudinal linear mixed regression models with a two-level structure with the observations (level 1) nested within the individuals (level 2), allowing for a random intercept and random slope on level 2. All models were inspected for multicollinearity, normal distribution of the residuals and homoscedasticity to fulfil model prerequisites. If residuals were not normally distributed, variables were log-transformed using the natural logarithm function (ln). First, a simple model including only the time variable (gestational age in weeks) as predictor was established, to see how the development over time could best be described (e.g., linear or quadratic). In the next step, all other variables were added to the model. Sedentary time and MVPA were included in one model, to obtain independent estimates. Both variables were converted before analysis to units of 10 min per day and were adjusted for accelerometer wear time. Light physical activity was not added in the model due to multicollinearity, as together with sedentary time and MVPA it makes up 100% of the measured daily physical activity. Further, maternal age (years), education, pre-pregnancy BMI (ln; kg/m^2^), randomisation group and country were included in the model. Offspring sex, which is associated with maternal postprandial glucose and risk of developing GDM [27], and severity of obesity were both considered as possible effect modifiers. Therefore, interactions between pre-pregnancy BMI/offspring sex and MVPA and sedentary time were added in additional models. Interactions with a *p*-value *<*0.10 were deemed statistically significant.

Mixed model analysis can be extended into a “hybrid” model, which produces separate estimates for within and between effects (within-between random effects model) [28]. Within-person effects reflect effects of longitudinal changes in sedentary time or MVPA on changes in the outcome variables during gestation, i.e., representing changes within one individual over time. Between-person effects reflect associations between individuals’ average differences in sedentary time or MVPA and average glucose/insulin parameters, i.e., representing differences between different individuals, averaged over the whole time period. Participants with missing observations were included on the assumption of “missing at random” (MAR).

As sensitivity analyses, mixed regression models were repeated on several adjusted datasets. Firstly, we conducted a complete case analysis, that is, excluding all participants with only two out of three accelerometer measurements. Secondly, as participants who developed GDM over the course of pregnancy were not allowed to partake in further OGTTs, analyses were rerun with only those who did not develop GDM. Analyses were further repeated with a categorised time variable, thus replacing gestational age in weeks by the three time points (<20 weeks, 24–28 weeks, 35–37 weeks). Lastly, analyses were repeated after imputing missing data by multivariate imputation by chained equations, taking the two-level structure of the data into account [29], using the “transform then impute” approach [30] for variables derived from other variables. Twenty imputed datasets were created and models were estimated in each separate dataset and subsequently pooled.

A two-tailed *p*-value of <0.05 was deemed significant for all analyses. All analyses were performed in R: A language and environment for statistical computing (version 3.6.1) [31]. Mixed model analyses were calculated using the lme4 package (version 1.1-21) [32]. Multiple imputation was performed using the mice package (version 3.6.0) [29]. Plots were produced using the packages dotwhisker (version 0.5.0) [33] and ggplot2 (version 3.2.1) [34].

## Results

### Study participants

**Fig. 1:**
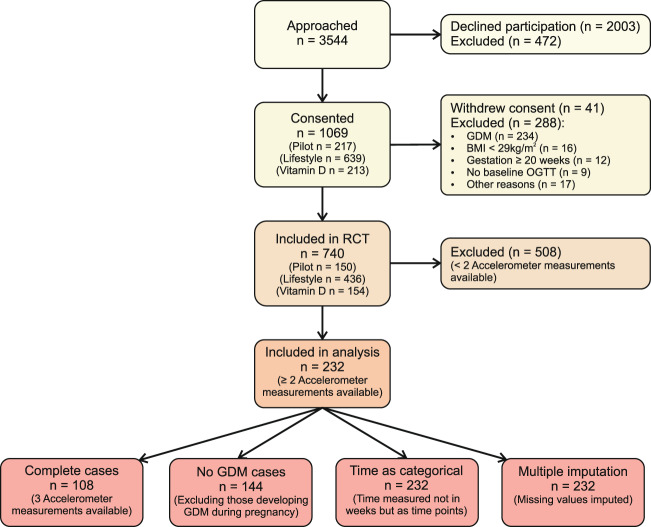
Flow diagram of study participants included in main analysis and sensitivity analyses. BMI Body mass index, GDM gestational diabetes mellitus, OGTT oral glucose tolerance test.

Figure 1 shows the flow chart of participants throughout the study. Of the original 740 women participating in the RCTs, 232 had sufficient accelerometer measurements to be included in this secondary analysis. In Table 1 baseline characteristics are compared between those included in the analysis versus those who were excluded. The groups were comparable, except for fewer Caucasian women and more women who were working in the included group.

**Table 1:** Baseline characteristics comparing included and excluded participants.

	Included (*n* = 232)	Excluded (*n* = 507)
Age, years (mean ± SD)	32.5 ± 5.2	31.8 ± 5.4
Pre-pregnancy BMI, kg/m^2^ (median (IQR))	32.5 (30.6–35.0)	32.9 (30.4–36.1)
Parity, multiparous (*n* (%))	120 (51.7)	252 (49.7)
Ethnicity, Caucasian (*n* (%))	189 (81.5)	450 (88.8)^a^
Education (*n* (%), total = 738)		
Low	27 (11.6)	63 (12.5)
Medium	75 (32.3)	159 (31.4)
High	130 (56.0)	284 (56.1)
Marital status, with partner (*n* (%), total = 738)	217 (93.5)	476 (94.1)
Occupational status, working (*n* (%))	200 (86.2)	374 (73.8)^a^
Smoking (*n* (%), total = 737)	33 (14.2)	89 (17.6)
Alcohol consumption (*n* (%), total = 731)	12 (5.2)	32 (6.4)
GDM diagnosis in pregnancy (*n* (%))	74 (33.5)	138 (35.8)
Gestational weight gain at 35–37 weeks, kg (mean ± SD)	8.0 ± 4.9	7.8 ± 4.8
Offspring sex, female (*n* (%), total = 668)	112 (49.3)	218 (49.4)

^a^represents a significant difference between included and excluded participants (*p*-value <0.05).

### Changes in sedentary time/MVPA and glucose/insulin parameters over time

Accelerometer data and blood parameters are presented separately for each time point in Table 2. Accelerometer wear time and time spent in MVPA significantly decreased over the course of pregnancy, while daily minutes spent sedentary remained constant. When expressed as percentage of wear time, sedentary time increased significantly from the first time point (<20 weeks) towards mid-pregnancy (24–28 weeks) from 69.7% to 71.5% but did not change further towards the end of pregnancy. Percentage of time spent in MVPA dropped significantly from mid to late pregnancy (4.0% to 3.7%).

**Table 2:** Sedentary time (ST), moderate-to-vigorous physical activity (MVPA) and metabolic parameters at three time points in pregnancy.

	*n*	T1 (<20 weeks)	*n*	T2 (24–28 weeks)	*n*	T3 (35–37 weeks)
Wear time, min/day (mean ± SD)	200	827.9 ± 87.6	196	802.5 ± 91.4^a^	176	789.1 ± 96.7^b^
Sedentary time, min/day (mean ± SD)	200	576.9 ± 99.6	196	575.3 ± 105.0	176	576.0 ± 94.7
Sedentary time, % of wear time (mean ± SD)	200	69.7 ± 9.5	196	71.5 ± 9.4^a^	176	73.0 ± 8.1
MVPA, min/day (median (IQR))	200	38.1 (23.7–53.0)	196	34.0 (21.0–50.8)^a^	176	29.6 (15.7–41.9)^b^
MVPA, % of wear time (median (IQR))	200	4.5 (2.9–6.3)	196	4.0 (2.5–6.6)	176	3.7 (2.0–5.0)^b^
Fasting glucose, mmol/l (mean ± SD)	232	4.7 ± 0.4	226	4.6 ± 0.4^a^	215	4.6 ± 0.4
1 h glucose, mmol/l (mean ± SD)	213	6.8 ± 1.4	223	7.5 ± 1.5^a^	194	8.0 ± 1.4^b^
2 h glucose, mmol/l (mean ± SD)	213	5.8 ± 1.2	224	6.2 ± 1.3^a^	194	6.6 ± 1.2^b^
Fasting insulin, mU/l (median (IQR))	227	13.1 (9.3–17.5)	226	14.7 (11.0–18.6)^a^	214	16.7 (12.7–23.1)^b^
1 h insulin, mU/l (median (IQR))	209	78.5 (53.5–151.1)	219	127.1 (68.7–176.5)^a^	194	178.9 (120.5–236.5)^b^
2 h insulin, mU/l (median (IQR))	208	56.5 (38.6–86.2)	220	71.2 (46.7–122.5)^a^	193	123.8 (67.4–185.8)^b^
HOMA-IR (median (IQR))	227	2.7 (1.9–3.6)	224	3.0 (2.2–3.9)^a^	213	3.4 (2.6–4.8)^b^
Stumvoll first phase (median (IQR))	206	1498.9 (1234.6–2035.4)	215	1820.2 (1353.7–2319.6)^a^	192	2592.5 (2031.7–3374.9)^b^
Stumvoll second phase (median (IQR))	206	385.0 (320.6–520.2)	215	468.8 (352.3–590.6)^a^	192	658.3 (522.0–846.5)^b^

^a^represents a significant change between T1 and T2 (*p*-value < 0.05).

^b^represents a significant change between T2 and T3 (*p*-value < 0.05).

Fasting glucose decreased slightly but significantly from <20 weeks until 24–28 weeks. Postprandial glucose, fasting and postprandial insulin, insulin resistance, and Stumvoll first and second phase all increased significantly throughout the whole pregnancy (Table 2).

### Longitudinal association between sedentary time/MVPA and glucose/insulin parameters

Main results are presented in Table 3. The development of Stumvoll first and second phase over the course of pregnancy can best be described quadratically, therefore gestational age has been added as a quadratic term for these two analyses.

**Table 3:** The longitudinal association between sedentary time (ST) and moderate-to-vigorous physical activity (MVPA) in pregnancy and metabolic parameters in obese women.

	Fasting glucose, mmol/l	Fasting insulin^a^, mU/l	HOMA-IR^a^	Stumvoll first phase^a^	Stumvoll second phase^a^
	Estimate (95% CI)	Estimate (95% CI)	Estimate (95% CI)	Estimate (95% CI)	Estimate (95% CI)
Sedentary time (within), 10 min/day	0.001 (-0.006, 0.007)	−0.002 (−0.010, 0.006)	−0.001 (−0.010, 0.007)	0.006* (0.000, 0.011)	0.006* (0.000, 0.011)
Sedentary time (between), 10 min/day	0.008** (0.002, 0.014)	0.011* (0.002, 0.019)	0.012** (0.004, 0.021)	0.008* (0.001, 0.014)	0.007* (0.001, 0.014)
MVPA (within)^a^, 10 min/day	0.019 (−0.035, 0.073)	0.037 (−0.028, 0.102)	0.042 (−0.028, 0.113)	0.018 (−0.026, 0.061)	0.016 (−0.026, 0.058)
MVPA (between)^a^, 10 min/day	0.052 (−0.011, 0.115)	−0.034 (−0.116, 0.048)	−0.022 (−0.110, 0.065)	−0.137*** (−0.210, −0.064)	−0.133*** (−0.202, −0.063)
*n* (observations)	232 (563)	232 (555)	232 (554)	226 (513)	226 (513)

Log transformed estimates can be interpreted as follows: when only the predictor variable is log-transformed, each 1% increase in the predictor variable increases the outcome variable by 1/100 units of the estimate. For example, a 1% increase in MVPA (between) is associated with a 0.0005 mmol/l increase in fasting glucose. When only the outcome variable is log-transformed, the estimate needs to be exponentiated and subtracted by 1. A one unit increase in outcome variable can then be interpreted as a percentage change of the outcome variable in the magnitude of the coefficient. For example, a one-unit increase in ST (between) is associated with a 1.2% (EXP(0.012)–1) increase in HOMA-IR. If both predictor and outcome variable are log-transformed, every 1% increase in the predictor variable is associated with an increase in percentage of the outcome variable in the magnitude of the coefficient. For example, a 1% increase in MVPA (between) is associated with a −0.133% change in Stumvoll second phase.

All analyses adjusted for gestational age, accelerometer wear time, maternal age, education, pre-pregnancy BMI, randomisation group, country, ST or MVPA.

^a^Natural log transformed values were used in the regression analyses.

*p < 0.05; **p < 0.01; ***p < 0.001.

Participants with on average more sedentary time had higher fasting glucose (Estimate: 0.008; 95% CI: 0.002, 0.014), increased ln fasting insulin (0.011; 0.002,0.019), increased ln HOMA-IR (0.012; 0.004, 0.021) and ln Stumvoll first and second phase (0.008; 0.001, 0.014 and 0.007; 0.001, 0.014), compared with those with less sedentary time (between participants effect). An increase in sedentary time throughout pregnancy (within participants effect) was associated with elevated ln Stumvoll first and second phase (both 0.006; 0.000, 0.011), but not with other outcomes. Participants with on average more time spent in ln MVPA had lower ln Stumvoll first and second phase (−0.137; −0.210, −0.064 and −0.133; −0.202, −0.063) (between participants effect), but changes in MVPA throughout pregnancy were not associated with Stumvoll first and second phase (within participants effect). MVPA was not significantly associated with other parameters.

The results presented here imply that if two comparable participants differ only in sedentary time (e.g., participant A is on average 60 min per day more sedentary than participant B throughout her pregnancy), participant A would have 0.048 mmol/l higher glucose levels, 6.8% higher fasting insulin, 7.5% higher HOMA-IR, 4.9% higher Stumvoll first phase and 4.3% higher Stumvoll second phase throughout pregnancy.

If participant A increased her sedentary behaviour over the course of pregnancy by 60 min, her Stumvoll first and second phase would increase by 3.6% each.

If participant A on average spent 10% more time in MVPA throughout her pregnancy compared to participant B (e.g., 44 min compared to 40 min per day), participant A would have 1.3% lower Stumvoll first phase and 1.3% lower Stumvoll second phase.

### Effect modification

When testing for effect modification by offspring sex, a statistically significant association was found between offspring sex and sedentary time. Other interactions were not significant. When stratifying results by offspring sex (Supplementary table 1), most significant associations were driven by women pregnant with male offspring. In participants pregnant with girls, there was a positive association between sedentary time (between participants) and fasting glucose, and a negative association between sedentary time (within participants) and fasting insulin and HOMA-IR.

When testing for effect modification by pre-pregnancy BMI, there was a significant positive interaction with fasting insulin and Stumvoll first and second phase. When stratifying results by median maternal BMI (<32.5 kg/m^2^ and ≥32.5 kg/m^2^), results indicated a stronger association between sedentary time and insulin secretion in the higher BMI group (results not shown).

### Sensitivity analyses

**Fig. 2:**
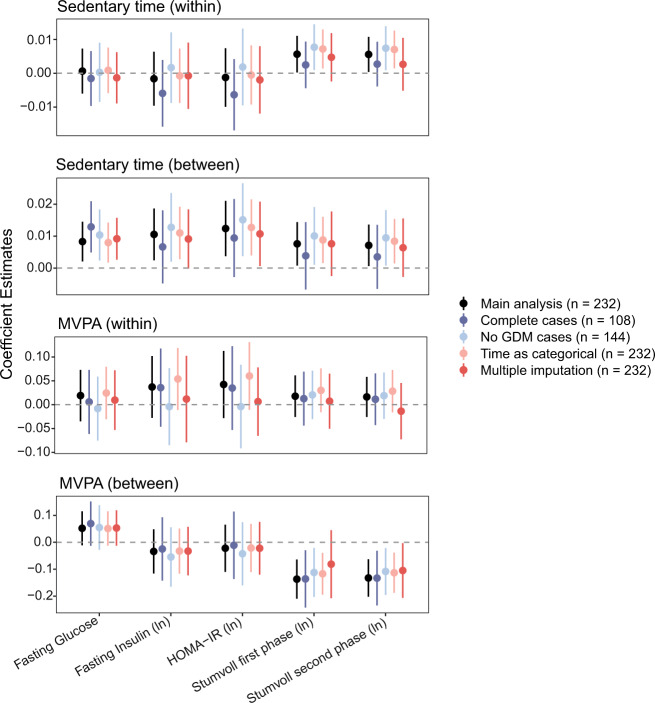
Graphical presentation of results of main analysis and sensitivity analyses showing coefficient estimates and 95% confidence intervals. GDM gestational diabetes mellitus, HOMA-IR Homeostatic model assessment for insulin resistance, MVPA moderate-to-vigorous physical activity.

In Figure 2 results of the main analysis and the sensitivity analyses are displayed graphically. Estimates and statistical significance of the sensitivity analyses in those without GDM and with the time variable categorised were similar to the main analysis. When analysing only complete cases participants, estimates were similar, but several associations were no longer statistically significant. After imputation, estimates again were similar but only the relationship between sedentary time (between-participants) and fasting glucose and insulin resistance remained statistically significant.

## Discussion

In this study we have shown that overweight and obese pregnant women with less sedentary time have more favourable glucose and insulin levels, insulin sensitivity and insulin secretion compared to women with more sedentary time. A reduction in sedentary time over the course of pregnancy is associated with decreased insulin secretion without a concurrent increase in glucose. This implies that next to promoting physical activity, reducing sedentary time should be encouraged in pregnancy. Our data also suggest that, as changes in behaviour throughout pregnancy have limited influence on maternal metabolism, interventions should target early pregnancy or pre-pregnancy. We found sex-differences, indicating that these associations were more pronounced in women pregnant with male offspring.

### Development of glucose/insulin parameters and sedentary time/MVPA over time

Our data showed a slight decrease in fasting glucose over the course of pregnancy, while fasting insulin, postprandial glucose and insulin, insulin resistance and secretion all increased progressively throughout pregnancy. These changes can all be attributed to physiological metabolic adaptations during pregnancy [1]. We also found a significant reduction in MVPA over time, which has been shown by other studies [35–37]. Time spent sedentary increased significantly during pregnancy, albeit only when adjusted for accelerometer wear time. While Di Fabio et al. also found a trend in increasing sedentary time with increasing gestation [37], others did not find any changes [35, 38, 39].

### Sedentary time and MVPA and glucose/insulin parameters

Our findings of a positive relationship between sedentary time (between estimate) and fasting glucose and insulin, insulin resistance and secretion are partly in line with the results of Wagnild et al., who found a significant association between objectively measured sedentary time and fasting and post-load glucose in those without GDM [40]. However, in the cross-sectional study of Gradmark et al. [41] and the longitudinal study of Nayak et al. [38], no associations between objectively measured sedentary time and either glucose or insulin sensitivity were found. Both studies comprised a relatively small sample size and therefore might have been underpowered to detect weak yet statistically significant associations.

We found a statistically significant positive association between MVPA and insulin secretion (between estimate). While outside of pregnancy very low values of insulin secretion are associated with reduced beta cell function, we think that in our study the reduction of Stumvoll first and second phase reflects a reduced need of insulin due to better insulin sensitivity and can therefore be seen as beneficial. This is endorsed by the fact that women in our study with GDM compared to those without had higher levels of insulin secretion (data not shown).

Other relationships with MVPA were not significant. This could be due to the participants in this study being overweight or obese, who might not provide enough variation in MVPA to detect significant relationships.

We found that differences in activity behaviour between women were more strongly associated with the glucose-insulin axis than changes in activity behaviour within a woman over time. We cannot preclude that unmeasured confounding (e.g., by dietary factors) might have influenced the between-participant estimates more than the within-participant estimates. Another explanation for our findings could be that our first measurement, which occurred at on average 15 weeks of gestation, might be too late to have an impact on the glucose-insulin axis within the remaining weeks of pregnancy. These findings are in line with a meta-analysis that found significant associations between physical activity in early or pre-pregnancy and reduced odds of GDM, with the highest reduction in odds with physical activity performed before pregnancy [42]. This is further corroborated by a review of Song et al. [43], that showed that only lifestyle interventions starting before 15 weeks of gestation are effective in the reduction of GDM risk. This could also explain the lack of effect of our intervention on metabolic outcomes. Overall, this suggests that changes in behaviour should take place before 15 weeks of gestation, thus in early pregnancy or even before pregnancy.

As our study only includes women with a BMI ≥ 29 kg/m^2^, results cannot be generalised to the general pregnant population. However, as the worldwide prevalence of obesity in women is predicted to surpass 21% by 2025 [44], it is essential to research how to reduce pregnancy complications in this high-risk population. Nevertheless, associations likely differ for obese and non-obese women. Even within our obese cohort, we found significant interactions between sedentary time and pre-pregnancy BMI, indicating stronger associations with increasing BMI. However, a larger sample size would be needed to stratify and look into specific BMI sub-groups separately.

We also found significant interactions between sedentary behaviour and foetal sex. The stratified results showed that most results were driven by women pregnant with male offspring. It has been shown that women pregnant with male offspring have poorer beta cell function and an increased risk of GDM, indicating foetal influence on the maternal metabolism [27, 45]. This supports the idea of lifestyle variables being associated differently with the maternal glucose-insulin axis, depending on foetal sex.

### Strengths and weaknesses

A weakness of this study is its observational design, which precludes us from studying causal effects. However, analysing the original RCT groups together as one cohort allowed us to firstly, pick up small, possibly relevant differences between individual participants that would be lost when comparing groups. Secondly, it allowed us to apply a longitudinal statistical method which incorporates repeated measurements over time into one analysis and produces separate estimates for changes in sedentary behaviour and MVPA within individual participants over time (within participants effect), as well as estimates that compare different participants with each other (between participants effect) [28].

Another weakness is the reduction in sample size compared to the original study (*n* = 740 versus *n* = 232). However, while reducing the number of included participants, it enabled us to study physical activity and sedentary behaviour in an objective way. The objective measurement of physical activity is especially important in our study of obese women, as individuals with higher body fat less accurately self-report physical activity than lean participants [46].

The objective measurement of physical activity with accelerometers is preferable to self-reported questionnaire data, which are subject to recall bias and are less robust when measuring light to moderate activity [47]. This accurate distinction between light physical activity and sedentary behaviour is essential. The time spent sedentary and in light physical activity and MVPA make up a person’s total day. For example, a reduction in sedentary time automatically results in an increase in MVPA or light physical activity. This is especially important in our study as we showed that reducing sedentary behaviour coinciding with unchanged MVPA, which consequently results in increased light physical activity, is associated with improved glucose and insulin parameters.

Light physical activity comprises exercises such as stretching or light weight training but mostly consists of non-exercise activities such as standing, light housework, walking or shopping. Non-exercise activities induce energy expenditure, defined as non-exercise activity thermogenesis (NEAT) [48]. NEAT accounts for the majority of a person’s daily variable energy expenditure [48] and can therefore play an important role in body weight regulation outside of pregnancy [49]. Our results support the notion that increased light physical activity and consequently increased NEAT also has positive effects in pregnancy.

A strength of our study is the analysis of sedentary behaviour independently of MVPA and vice versa. Adjusting analyses for both sedentary behaviour and MVPA is essential to tease apart the effect of reducing sedentary behaviour from increasing MVPA, as it has been shown that individuals with increased sedentary time have a higher risk of adverse health outcomes, even when achieving recommended physical activity levels [15].

The sensitivity analyses, which were performed to test the robustness of the results, also strengthen our study. The sensitivity analyses with gestational age as categorical variable and only participants without GDM showed results and significances very similar to the original analysis. The complete case and multiple imputation analyses showed similar albeit mostly non-significant estimates. This could be due to the wider confidence intervals in those two analyses, caused by the reduced sample size in the complete case analysis (*n* = 144 versus *n* = 232) and by the added random error in the multiple imputation model.

### Practical implications

Our results imply that reducing sedentary behaviour in obese pregnant women might be more relevant for metabolic health than increasing physical activity. It would also be beneficial for neonatal outcomes, since we previously found that a reduction of neonatal adiposity was mediated by a reduction of sedentary time [18]. In the general population, recent public health guidelines have also stressed the importance of reducing sedentary time in addition to physical activity [50]. Specific guidelines in pregnancy on sedentary behaviour might further help health care providers, especially since they often struggle with giving advice on physical activity [51]. Furthermore, focussing on changing sedentary behaviour might also be easier for obese women than increasing physical activity levels alone.

Future intervention studies, focussing on reducing sedentary behaviour by encouraging light physical activity and breaking up sitting time, are needed. Those studies should also include leaner women to compare effects between BMI groups. Furthermore, while the clinical relevance of our results appears to be limited for the mother, the relevance for the offspring remains unclear. Future studies are needed to determine the clinical relevance of reduced sedentary time in pregnancy on mother and offspring.

In conclusion, our study underlines the importance of sedentary behaviour in pregnancy and the need for further studies focussing on interventions in early and pre-pregnancy.

## Supplementary information

Supplementary Table 1

## Data Availability

Data are available from the corresponding author on reasonable request.
