# Supplementary material for: Less sedentary time is associated with a more favourable glucose-insulin axis in obese pregnant women—a secondary analysis of the DALI study
Source: Int J Obes (Lond). 2020 Jul 13;45(2):296–307. doi: 10.1038/s41366-020-0639-y (PMC7840500; doi:10.1038/s41366-020-0639-y)
Supplement: Supplementary file 1 — Supplementary Table 1 [file 41366_2020_639_MOESM1_ESM.pdf]

Supplementary Table 1: The longitudinal association between sedentary time (ST) and moderate-to-vigorous physical activity (MVPA) in pregnancy and metabolic parameters stratified by offspring sex

	Fasting glucose, *mmol/l* *Estimate (95% CI)*	Fasting insulin[a], *mU/l* *Estimate (95% CI)*	HOMA-IR[a] *Estimate (95% CI)*	Stumvoll first phase[a] *Estimate (95% CI)*	Stumvoll second phase[a] *Estimate (95% CI)*
**Female offspring**					
Sedentary time (within), *10 min/day*	-0.005 (-0.014, 0.005)	-0.015** (-0.026, -0.004)	-0.016** (-0.028, -0.005)	-0.003 (-0.011, 0.005)	-0.002 (-0.010, 0.005)
Sedentary time (between), *10 min/day*	0.010** (0.003, 0.018)	0.009 (-0.002, 0.020)	0.011 (-0.001, 0.023)	0.008 (-0.001, 0.017)	0.007 (-0.001, 0.016)
MVPA (within)[a], *10 min/day*	0.013 (-0.067, 0.092)	0.035 (-0.054, 0.123)	0.042 (-0.052, 0.137)	-0.011 (-0.073, 0.050)	-0.013 (-0.072, 0.047)
MVPA (between)[a], *10 min/day*	0.030 (-0.057, 0.117)	-0.027 (-0.150, 0.095)	-0.018 (-0.149, 0.113)	-0.068 (-0.170, 0.034)	-0.065 (-0.163, 0.033)
n (observations)	112 (276)	112 (272)	112 (272)	112 (255)	112 (255)
**Male offspring**					
Sedentary time (within), *10 min/day*	0.007 (-0.003, 0.017)	0.010 (-0.002, 0.021)	0.011 (-0.002, 0.024)	0.014*** (0.006, 0.021)	0.013*** (0.006, 0.021)
Sedentary time (between), *10 min/day*	0.010 (0.000, 0.020)	0.016* (0.003, 0.029)	0.018** (0.005, 0.032)	0.011* (0.001, 0.022)	0.011* (0.001, 0.020)
MVPA (within)[a], *10 min/day*	0.022 (-0.053, 0.097)	0.029 (-0.065, 0.123)	0.035 (-0.068, 0.137)	0.044 (-0.016, 0.104)	0.042 (-0.016, 0.099)
MVPA (between)[a], *10 min/day*	0.084 (-0.009, 0.177)	-0.069 (-0.182, 0.043)	-0.054 (-0.175, 0.067)	-0.216*** (-0.316, -0.117)	-0.210*** (-0.305, -0.115)
n (observations)	115 (276)	115 (272)	115 (271)	109 (247)	109 (247)

*$p < 0.05$; **$p < 0.01$; ***$p < 0.001$

[a] Natural log transformed values were used in the regression analyses

All analyses adjusted for gestational age, accelerometer wear time, maternal age, education, pre-pregnancy BMI, randomization group, country, ST or MVPA

Log transformed estimates can be interpreted as follows: When only the predictor variable is log-transformed, each 1% increase in the predictor variable increases the outcome variable by 1/100 units of the estimate. For example, a 1% increase in MVPA (between) in those with male offspring is associated with a 0.0008 mmol/l increase in fasting glucose. When only the outcome variable is log-transformed, the estimate needs to be exponentiated and subtracted by 1. A one unit increase in outcome variable can then be interpreted as a percentage change of the outcome variable in the magnitude of the coefficient. For example, a one-unit increase in ST (between) in those with male offspring is associated with a 1.8% (EXP(0.018) – 1) increase in HOMA-IR. If both predictor and outcome variable are log-transformed, every 1% increase in the predictor variable is associated with an increase in percentage of the outcome variable in the magnitude of the coefficient. For example, a 1% increase in MVPA (between) in those with male offspring is associated with a -0.210% change in Stumvoll second phase.
